# Retracted: Anaerobic Treatment of Palm Oil Mill Effluent in Pilot-Scale Anaerobic EGSB Reactor

**DOI:** 10.1155/2018/5284306

**Published:** 2018-03-01

**Authors:** BioMed Research International

BioMed Research International has retracted the article titled “Anaerobic Treatment of Palm Oil Mill Effluent in Pilot-Scale Anaerobic EGSB Reactor” [1]. The article was found to be a dual publication of the following published article: “Jin Wang, Qaisar Mahmood, Jiang-Ping Qiu, et al., “Zero Discharge Performance of an Industrial Pilot-Scale Plant Treating Palm Oil Mill Effluent,” BioMed Research International, vol. 2015, Article ID 617861, 9 pages, 2015. doi:10.1155/2015/617861.”
